# Cyano­methanaminium perchlorate

**DOI:** 10.1107/S1600536812047782

**Published:** 2012-11-30

**Authors:** Jing Quan

**Affiliations:** a Department of Applied Chemistry, Nanjing College of Chemical Technology, Nanjing 210048, People’s Republic of China

## Abstract

In the crystal of the title salt, C_2_H_5_N_2_^+^·ClO_4_^−^, the cations and anions are connected *via* N—H⋯O and C—H⋯O hydrogen bonds, forming a three-dimensional network.

## Related literature

For general background, see: Haertling (1999 ▶); Homes *et al.* (2001 ▶). For a related structure, see: Han & Zhang (2010 ▶).
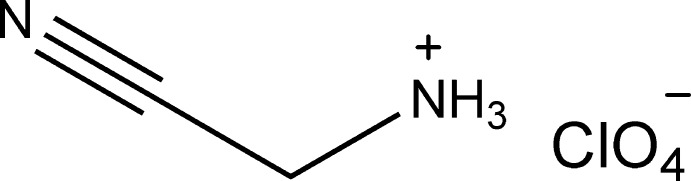


## Experimental

### 

#### Crystal data

C_2_H_5_N_2_^+^·ClO_4_^−^
*M* *_r_* = 156.53
Orthorhombic, 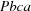

*a* = 9.908 (2) Å
*b* = 10.398 (2) Å
*c* = 11.176 (2) Å
*V* = 1151.4 (4) Å^3^
*Z* = 8
Mo *K*α radiation
μ = 0.61 mm^−1^
*T* = 293 K
0.20 × 0.19 × 0.18 mm

#### Data collection

Rigaku Mercury2 diffractometer
Absorption correction: multi-scan (*CrystalClear*; Rigaku, 2005 ▶) *T*_min_ = 0.88, *T*_max_ = 0.90
10965 measured reflections
1321 independent reflections
1151 reflections with *I* > 2σ(*I*)
*R*_int_ = 0.044

#### Refinement

*R*[*F* ^2^ > 2σ(*F* ^2^)] = 0.034
*wR*(*F* ^2^) = 0.096
*S* = 1.12
1321 reflections
84 parameters
H-atom parameters constrained
Δρ_max_ = 0.40 e Å^−3^
Δρ_min_ = −0.37 e Å^−3^

### 

Data collection: *CrystalClear* (Rigaku, 2005 ▶); cell refinement: *CrystalClear*; data reduction: *CrystalClear*; program(s) used to solve structure: *SHELXTL* (Sheldrick, 2008 ▶); program(s) used to refine structure: *SHELXTL*; molecular graphics: *SHELXTL*; software used to prepare material for publication: *SHELXTL*.

## Supplementary Material

Crystal structure: contains datablock(s) I, global. DOI: 10.1107/S1600536812047782/xu5654sup1.cif

Structure factors: contains datablock(s) I. DOI: 10.1107/S1600536812047782/xu5654Isup2.hkl

Supplementary material file. DOI: 10.1107/S1600536812047782/xu5654Isup3.cml

Additional supplementary materials:  crystallographic information; 3D view; checkCIF report

## Figures and Tables

**Table 1:** Hydrogen-bond geometry (Å, °)

*D*—H⋯*A*	*D*—H	H⋯*A*	*D*⋯*A*	*D*—H⋯*A*
N1—H1*C*⋯O1^i^	0.89	2.10	2.920 (2)	152
N1—H1*D*⋯O4	0.89	2.03	2.919 (2)	175
N1—H1*E*⋯O2^ii^	0.89	2.10	2.914 (3)	152
C1—H1*A*⋯O1^iii^	0.97	2.49	3.456 (3)	172
C1—H1*B*⋯O3^iv^	0.97	2.57	3.532 (3)	169

Symmetry codes: (i) 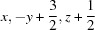; (ii) 
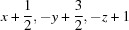
; (iii) 
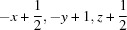
; (iv) 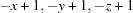.

**Fig. 1.**
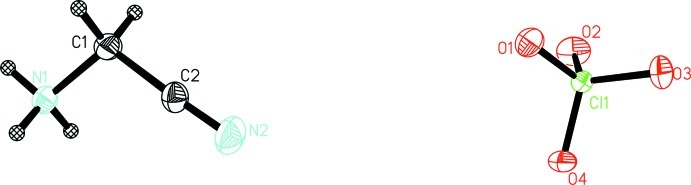
Perspective structure of the title compound with displacement ellipsoids drawn at the 50% probability level. The dashed line indicates N—H···O hydrogen bond.

**Fig. 2.**
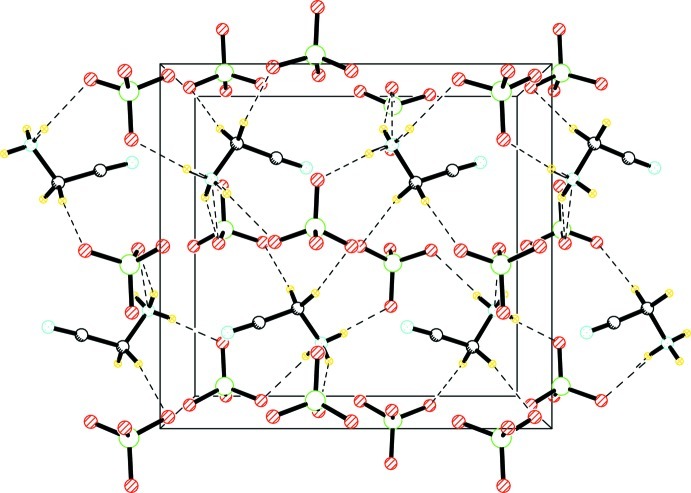
The crystal packing of the title compound viewed along thea axis showing the hydrogen bondings network.

## supplementary crystallographic information

### Comment

At present, much attention in ferroelectric material field is focused on developing ferroelectric pure organic or inorganic compounds (Haertling, 1999; Homes *et al.*, 2001). In order to find more dielectric ferroelectric materials, we investigate the physical properties of the title compound (Fig. 1). The dielectric constant of the title compound as a function of temperature indicates that the permittivity is basically temperature-independent (dielectric constant equaling to 3.7 to 5.2), suggesting that this compound should be not a real ferroelectrics or there may be no distinct phase transition occurred within the measured temperature range. Similarly, below the melting point (453 K) of the compound, the dielectric constant as a function of temperature also goes smoothly, and there is no dielectric anomaly observed (dielectric constant equaling to 3.7 to 5.2). Herein, we report the synthesis and crystal structure of the title compound.

Molecules of the title compound have normal geometric parameters. The bond lengths and angles are within their normal ranges (Han & Zhang, 2010). As can be seen from the packing diagram (Fig. 2), molecules are connected *via* intermolecular N—H···O and C—H···O hydrogen bonds to form a three dimensional network.

### Experimental

A mixture of aminoacetonitrile hydrochloride (0.095 g, 0.01 mol) and perchloric acid (1.40 g, 0.01 mol) in methanol (20 ml) was stirred until clear. After several days, the title compound was formed and recrystallized from solution to afford colourless prismatic crystals suitable for X-ray analysis.

### Refinement

H atoms were positioned geometrically and refined using a riding model with C—H = 0.97 and N—H = 0.89 Å, *U*_iso_(H) = 1.2*U*_eq_(C) and 1.5*U*_eq_(N).

### Crystal data

**Table:**

C_2_H_5_N_2_^+^·ClO_4_^−^	*F*(000) = 640
*M**_r_* = 156.53	*D*_x_ = 1.806 Mg m^−^^3^
Orthorhombic, *P**b**c**a*	Mo *K*α radiation, λ = 0.71073 Å
Hall symbol: -P 2ac 2ab	Cell parameters from 1321 reflections
*a* = 9.908 (2) Å	θ = 2.6–27.5°
*b* = 10.398 (2) Å	µ = 0.61 mm^−^^1^
*c* = 11.176 (2) Å	*T* = 293 K
*V* = 1151.4 (4) Å^3^	Prism, colorless
*Z* = 8	0.20 × 0.19 × 0.18 mm

### Data collection

**Table:**

Rigaku Mercury2 diffractometer	1321 independent reflections
Radiation source: fine-focus sealed tube	1151 reflections with *I* > 2σ(*I*)
Graphite monochromator	*R*_int_ = 0.044
Detector resolution: 13.6612 pixels mm^-1^	θ_max_ = 27.5°, θ_min_ = 3.4°
CCD_Profile_fitting scans	*h* = −12→12
Absorption correction: multi-scan (*CrystalClear*; Rigaku, 2005)	*k* = −13→13
*T*_min_ = 0.88, *T*_max_ = 0.90	*l* = −14→14
10965 measured reflections	

### Refinement

**Table:**

Refinement on *F*^2^	Secondary atom site location: difference Fourier map
Least-squares matrix: full	Hydrogen site location: inferred from neighbouring sites
*R*[*F*^2^ > 2σ(*F*^2^)] = 0.034	H-atom parameters constrained
*wR*(*F*^2^) = 0.096	*w* = 1/[σ^2^(*F*_o_^2^) + (0.0373*P*)^2^ + 0.8373*P*] where *P* = (*F*_o_^2^ + 2*F*_c_^2^)/3
*S* = 1.12	(Δ/σ)_max_ = 0.002
1321 reflections	Δρ_max_ = 0.40 e Å^−^^3^
84 parameters	Δρ_min_ = −0.37 e Å^−^^3^
0 restraints	Extinction correction: *SHELXTL* (Sheldrick, 2008), Fc^*^=kFc[1+0.001xFc^2^λ^3^/sin(2θ)]^-1/4^
Primary atom site location: structure-invariant direct methods	Extinction coefficient: 0.072 (3)

### Special details

**Table:**

Geometry. All e.s.d.'s (except the e.s.d. in the dihedral angle between two l.s. planes) are estimated using the full covariance matrix. The cell e.s.d.'s are taken into account individually in the estimation of e.s.d.'s in distances, angles and torsion angles; correlations between e.s.d.'s in cell parameters are only used when they are defined by crystal symmetry. An approximate (isotropic) treatment of cell e.s.d.'s is used for estimating e.s.d.'s involving l.s. planes.
Refinement. Refinement of *F*^2^ against ALL reflections. The weighted *R*-factor *wR* and goodness of fit *S* are based on *F*^2^, conventional *R*-factors *R* are based on *F*, with *F* set to zero for negative *F*^2^. The threshold expression of *F*^2^ > σ(*F*^2^) is used only for calculating *R*-factors(gt) *etc*. and is not relevant to the choice of reflections for refinement. *R*-factors based on *F*^2^ are statistically about twice as large as those based on *F*, and *R*- factors based on ALL data will be even larger.

### Fractional atomic coordinates and isotropic or equivalent isotropic displacement parameters (Å^2^)

**Table:**

	*x*	*y*	*z*	*U*_iso_*/*U*_eq_	
Cl1	0.24234 (4)	0.55298 (4)	0.39030 (4)	0.0262 (2)	
N1	0.38874 (19)	0.80122 (18)	0.60010 (15)	0.0338 (4)	
H1C	0.3343	0.8461	0.6478	0.051*	
H1D	0.3423	0.7726	0.5374	0.051*	
H1E	0.4558	0.8514	0.5750	0.051*	
N2	0.5768 (2)	0.7581 (2)	0.85728 (18)	0.0503 (6)	
O1	0.30000 (17)	0.51248 (16)	0.27907 (14)	0.0432 (4)	
O2	0.10533 (16)	0.50926 (18)	0.39645 (16)	0.0485 (5)	
O3	0.31737 (19)	0.50020 (19)	0.48743 (16)	0.0521 (5)	
O4	0.24459 (16)	0.69160 (15)	0.39638 (15)	0.0414 (5)	
C1	0.4448 (2)	0.6911 (2)	0.6667 (2)	0.0354 (5)	
H1A	0.3718	0.6343	0.6900	0.042*	
H1B	0.5049	0.6432	0.6147	0.042*	
C2	0.5189 (2)	0.7311 (2)	0.77361 (19)	0.0335 (5)	

### Atomic displacement parameters (Å^2^)

**Table:**

	*U*^11^	*U*^22^	*U*^33^	*U*^12^	*U*^13^	*U*^23^
Cl1	0.0279 (3)	0.0258 (3)	0.0249 (3)	0.00019 (17)	−0.00002 (18)	−0.00046 (17)
N1	0.0368 (10)	0.0363 (10)	0.0283 (9)	−0.0023 (7)	−0.0062 (7)	−0.0005 (7)
N2	0.0541 (12)	0.0613 (15)	0.0354 (11)	−0.0057 (11)	−0.0128 (9)	−0.0011 (10)
O1	0.0516 (10)	0.0449 (9)	0.0331 (9)	0.0040 (8)	0.0122 (7)	−0.0064 (7)
O2	0.0319 (9)	0.0492 (10)	0.0643 (12)	−0.0084 (7)	0.0085 (8)	−0.0033 (8)
O3	0.0638 (12)	0.0515 (10)	0.0409 (10)	0.0079 (9)	−0.0182 (9)	0.0077 (8)
O4	0.0529 (11)	0.0247 (9)	0.0466 (11)	0.0000 (6)	−0.0076 (8)	−0.0046 (6)
C1	0.0415 (11)	0.0306 (11)	0.0341 (11)	−0.0017 (9)	−0.0089 (10)	−0.0009 (9)
C2	0.0308 (10)	0.0386 (11)	0.0310 (11)	−0.0019 (9)	−0.0016 (8)	0.0028 (9)

### Geometric parameters (Å, º)

**Table:**

Cl1—O3	1.4256 (17)	N1—H1D	0.8900
Cl1—O1	1.4314 (16)	N1—H1E	0.8900
Cl1—O2	1.4333 (17)	N2—C2	1.133 (3)
Cl1—O4	1.4431 (16)	C1—C2	1.462 (3)
N1—C1	1.475 (3)	C1—H1A	0.9700
N1—H1C	0.8900	C1—H1B	0.9700
			
O3—Cl1—O1	109.87 (11)	H1C—N1—H1E	109.5
O3—Cl1—O2	109.59 (12)	H1D—N1—H1E	109.5
O1—Cl1—O2	109.04 (11)	C2—C1—N1	112.37 (18)
O3—Cl1—O4	109.92 (11)	C2—C1—H1A	109.1
O1—Cl1—O4	109.18 (10)	N1—C1—H1A	109.1
O2—Cl1—O4	109.22 (10)	C2—C1—H1B	109.1
C1—N1—H1C	109.5	N1—C1—H1B	109.1
C1—N1—H1D	109.5	H1A—C1—H1B	107.9
H1C—N1—H1D	109.5	N2—C2—C1	177.8 (2)
C1—N1—H1E	109.5		

### Hydrogen-bond geometry (Å, º)

**Table:**

*D*—H···*A*	*D*—H	H···*A*	*D*···*A*	*D*—H···*A*
N1—H1*C*···O1^i^	0.89	2.10	2.920 (2)	152
N1—H1*D*···O4	0.89	2.03	2.919 (2)	175
N1—H1*E*···O2^ii^	0.89	2.10	2.914 (3)	152
C1—H1*A*···O1^iii^	0.97	2.49	3.456 (3)	172
C1—H1*B*···O3^iv^	0.97	2.57	3.532 (3)	169

Symmetry codes: (i) *x*, −*y*+3/2, *z*+1/2; (ii) *x*+1/2, −*y*+3/2, −*z*+1; (iii) −*x*+1/2, −*y*+1, *z*+1/2; (iv) −*x*+1, −*y*+1, −*z*+1.
